# Pomegranate juice to reduce fecal calprotectin levels in inflammatory bowel disease patients with a high risk of clinical relapse: Study protocol for a randomized controlled trial

**DOI:** 10.1186/s13063-019-3321-8

**Published:** 2019-06-06

**Authors:** Eleonora Scaioli, Andrea Belluzzi, Luigi Ricciardiello, Daniele Del Rio, Enrica Rotondo, Pedro Mena, Eleonora Derlindati, Francesca Danesi

**Affiliations:** 1 Gastroenterological Unit, Department of Medical and Surgical Sciences, University of Bologna, Via Massarenti 9, 40138 Bologna, Italy; 2 Gastroenterological Unit, St. Orsola-Malpighi Hospital, Via Massarenti 9, 40138 Bologna, Italy; 3 Department of Veterinary Science, University of Parma, Strada del Taglio, 10, 43126 Parma, Italy; 4 Human Nutrition Unit, Department of Agricultural and Food Sciences, Campus of Food Science, University of Bologna, Piazza Goidanich, 60, 47521 Cesena, FC Italy; 5 Human Nutrition Unit, Department of Food and Drug, University of Parma, Medical School, Building C, Via Volturno 39, 43125 Parma, Italy; 6 Endocrinology and Metabolism Unit, Department of Medicine and Surgery, University of Parma, Via Gramsci 14, 43126 Parma, Italy

**Keywords:** Ellagitannins, Pomegranate juice, *Punica granatum* L., Inflammation, Fecal calprotectin, IBD, Inflammatory bowel disease, Crohn’s disease, Ulcerative colitis

## Abstract

**Background:**

Inflammatory bowel disease (IBD) is a chronic condition characterized by recurrent episodes of intestinal inflammation and is thought to be related to an autoimmune reaction to genetic and environmental factors. Although evidence indicates that a polyphenolic-rich diet plays an important role in modulating aspects of chronic inflammation, few studies have focused on the effect of ellagitannin (ET)-rich food consumption on long-term remission maintenance in IBD patients with a high risk of clinical relapse. Therefore, we hypothesize that supplementation with a pomegranate juice, a naturally rich source of ETs, could significantly modulate the markers of mucosal and systemic inflammation relative to a control group receiving a placebo.

**Methods/design:**

This double-blind, randomized controlled trial includes patients with IBD involving the colorectum who have been in stable therapy for at least the three previous months and have a high risk of clinical relapse. Participants are randomly allocated to one of two groups: active supplementation (125 mL of cv. Wonderful pomegranate juice) or placebo (125 mL) taken twice daily for 12 weeks. The primary outcome is changes in the fecal neutrophil-derived protein calprotectin, a surrogate marker of mucosal improvement, between the two groups from baseline to 12 weeks later. The secondary outcomes include transcriptomic changes in peripheral blood mononuclear cells and intestinal biopsies and changes in circulating inflammatory markers and trimethylamine-*N*-oxide levels. Pomegranate ET-derived metabolites are identified and quantified in plasma and urine samples.

**Discussion:**

The results will provide information on the possible reduction of fecal calprotectin levels following the consumption of pomegranate juice. The findings will also show the in vivo metabolism of pomegranate ETs. Finally, the effect of 12-week pomegranate juice consumption on local and systemic inflammatory markers will be elucidated, which will likely provide additional insights into the maintenance of remission in IBD patients.

**Trial registration:**

ClinicalTrials.gov, NCT03000101. Registered on 21 December 2016.

**Electronic supplementary material:**

The online version of this article (10.1186/s13063-019-3321-8) contains supplementary material, which is available to authorized users.

## Background

Inflammatory bowel disease (IBD) has a multifactorial etiology and is thought to be related to a combination of individual genetic susceptibility and environmental triggers that stimulate an inflammatory response. Ulcerative colitis (UC) and Crohn’s disease (CD), the two main types of IBD, are chronic conditions that usually follow a relapsing clinical course with phases of remission that alternate with periods of active inflammation [1]. Generally, after medically induced remission, about half of the patients who do not receive therapy will relapse in the following year [2].

Fecal calprotectin (FC), a calcium-binding protein derived from neutrophils, monocytes, and reactive macrophages [3], has been shown to be a useful marker for defining subclinical persistent mucosal inflammation in IBD and has demonstrated a positive linear correlation with both endoscopic and histological activity [4–8]. In particular, a FC ≥ 100 μg/g intercepts a subgroup of patients in clinical remission with a high risk of relapse [9–11]. However, a combination of this FC marker and other inflammatory response markers and disease-specific activity indices can increase diagnostic accuracy in reference to FC elevation [12–14].

Several relevant molecular mechanisms contribute to chronic inflammation in IBD. The involved mechanisms appear to be related to the dysregulation of selected signaling pathways in the colon, such as nuclear factor κ light-chain-enhancer of activated B cells (NF-κB), c-Jun N-terminal kinase (JNK), extracellular signal-regulated kinase (ERK), and signal transducer and activator of transcription 3 (STAT3) phosphorylation [15, 16]. In addition, relevant molecular processes associated with clinically quiescent inflammatory states in tissues other than the colon can be studied by analyzing peripheral blood gene expression patterns to identify novel transcriptional signatures related to incoming relapses [17].

Critical goals for IBD are to preserve a condition of clinical remission. Diet may be an important factor in the maintenance of long-term diminution of disease severity. In particular, a diet rich in phytochemicals, such as polyphenols, could represent a strategy in the modulation of chronic intestinal inflammation [18]. In particular, strong evidence indicates that polyphenol-rich pomegranate supplementation exerts anti-inflammatory effects that may ameliorate the symptoms of chronic inflammatory diseases and IBD in rodent models [15], probably due to the formation of ellagitannin (ET) metabolites in the colon. ETs are polyphenols present in berries (cloudberry, raspberry, rosehip, strawberry, and sea buckthorn), pomegranate, walnuts and some other nuts, and oak-aged wines [19]. Upon hydrolysis in the gut, ETs undergo lactonization to produce ellagic acid, which is converted into urolithins by the microbiota [20]. These metabolites occur at relatively high concentrations in the colon (6–168 μM urolithin A, the most abundant metabolite, in rodents and pigs) [21] and can exert anti-inflammatory effects at both local (colon) and systemic levels [22–25]. Despite these promising premises, few studies have explored the potential benefits of ET-rich food on IBD in humans.

In the present exploratory study, patients with IBD in stable clinical remission with increasing inflammation identified by increased FC values were asked to consume an ET-rich pomegranate juice in order to test systemic and mucosal changes in biochemical and molecular inflammatory response markers relative to a control group receiving a placebo.

## Methods/design

### Objectives

The main purpose of the intervention trial is to assess the possible effect of pomegranate juice consumption on reducing FC levels in volunteers suffering from IBD with a high risk of clinical relapse. In detail, the objectives of this intervention are as follows:

(i) assessing the changes in FC levels in the two groups (active treatment, control) from baseline to 12 weeks later (primary outcome)
(ii) investigating the systemic and mucosal modifications of selected biochemical and molecular inflammatory response markers in the two groups after 12 weeks of the intervention compared with baseline (secondary outcomes)
(iii) evaluating circulating and urinary ET-derived metabolites from regular pomegranate juice consumption in the two groups before and after the intervention

### Protocol and study design

**Fig. 1:**
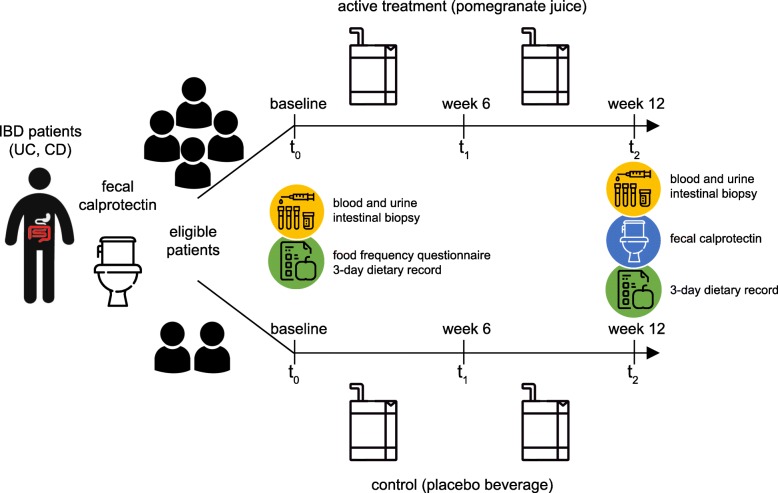
Scheme of the study design. *IBD* inflammatory bowel disease, *UC* ulcerative colitis, *CD* Crohn’s disease

**Fig. 2:**
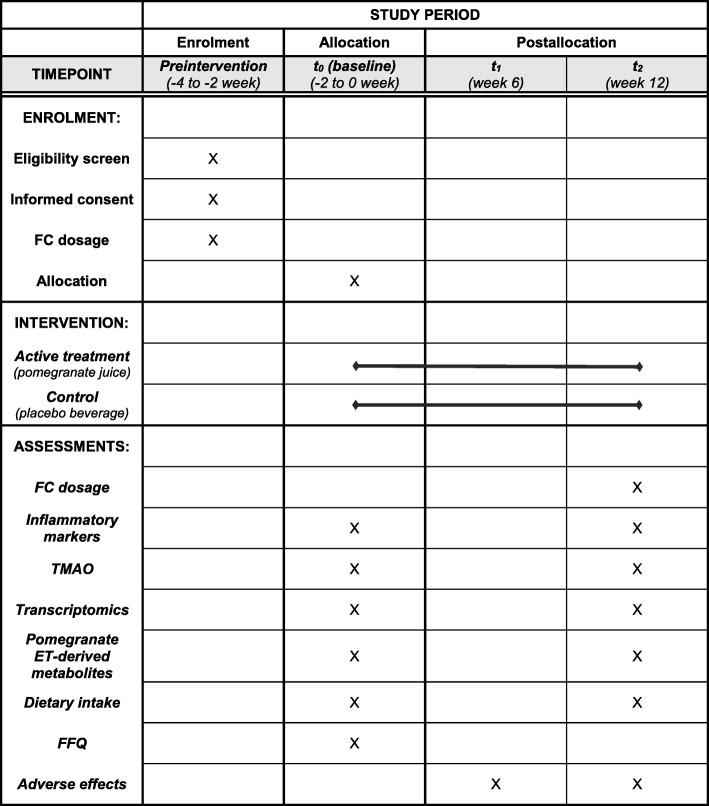
Schedule of enrolment, intervention, and assessments in the study. *FC* fecal calprotectin, *TMAO* trimethylamine *N*-oxide, *ET* ellagitannin, *FFQ* food frequency questionnaire

An overview of the study design is reported in Fig. 1 and in the SPIRIT chart [26] (Fig. 2) (see Additional file 1 for the SPIRIT checklist). This intervention trial with a randomized and controlled design is a two-armed study with parallel groups. The effects of active treatment (pomegranate juice) are compared with those of a control (placebo), and patients with IBD, either UC or CD, in stable clinical remission are randomly assigned to two groups. Subjects are given 125 mL twice daily of treatment or placebo for 12 weeks. Participants are asked to collect their stool sample prior to the pre-intervention visit and at the end of the intervention (t_2_) if they are successfully enrolled. Before (t_0_) and after the intervention (t_2_), biological samples (blood, urine, and intestinal biopsies) are collected. An intermediate visit takes place 6 weeks after test visit 1 (t_1_) to assess any adverse effects and changes to medications or health status to ensure that participants are still suitable for the study.

During the intervention, subjects are asked to maintain their dietary habits with a limited daily intake (150 mL, approximately one glass) of 100% fruit juices, fruit nectars, and fruit juice drinks. The consumption of fruits, jams, juices, and seeds rich in ETs (e.g., raspberries, blackberries, strawberries, pomegranate, walnuts, and chestnuts) or dietary supplements containing polyphenols is not allowed during the study.

Some minimal dietary restrictions are introduced one day before sampling times. During the 24 h prior to the sample collection period, subjects are required to abstain from eating meals containing trimethylamine *N*-oxide (TMAO; e.g., fish and shellfish) and its dietary precursors, choline (eggs), and carnitine (red and processed meat); dietary restrictions are extended to dairy products, poultry, and foods, such as whole foods, which are thought to affect TMAO metabolism [27]. A sample meal plan is as follows: coffee or tea with non-whole-grain cookies for breakfast, tomato pizza or flatbread and vegetables for lunch, pasta or rice and vegetables at dinner, and one or two fruits low in ETs as snacks.

### Intervention

A 125-mL dose of either pomegranate juice or placebo is given twice a day.

The placebo formulation (water, sucrose, and citric acid monohydrate, previously used in a study with healthy volunteers with no clinically relevant effects [28]) is designed to match the moisture (percentage), carbohydrate (percentage), total soluble solids content (degrees Brix), titratable acidity (percentage), and energy (kcal) of pomegranate juice. A daily dose of pomegranate juice provides an estimated 300 mg of ETs or higher [29]; ETs are absent in the placebo.

Pure pomegranate juices from three different cultivars (Wonderful, Hicaznar, and Mollar de Elche) and pomegranate juices from concentrate at three different dilutions (50%, 75%, and 100%) were tested by a panel of 20 healthy persons before the study. A cv. Wonderful 100% pomegranate juice (Gat Foods; M.P. Hefer, Israel) was chosen based on the sensory quality, including appearance and flavor (taste and aroma), of the different test products.

Placebo and pomegranate juice are supplied by Conserve Italia (Bologna, Italy) in a white juice box holding 125 mL of beverage with a drinking straw (Tetra Brik® Aseptic, Tetra Pak, Lund, Sweden). Beverages are packaged indistinguishably and labeled with the participant’s ID number. The beverages are provided to all participants in two 6-week supplies at the end of the allocation visit (t_0_) and the intermediate visit (t_1_). Volunteers are advised to shake the drink well before opening it and to consume it as a mid-morning or mid-afternoon snack.

### Participants

Eligible patients are asymptomatic subjects aged 18–80 years with UC or CD (the latter mainly involving sigmoid colon and rectum) in stable clinical remission (SCCAI, simple clinical colitis activity index = 0 [30] for UC patients; CDAI, CD activity index < 150 [31] for CD patients) for at least 3 months with FC levels higher than 100 μg/g [9–11]. Concomitant stable therapies for UC (mesalamine, immunomodulators, and/or biological drugs) without modifications in the previous 3 months are allowed. The exclusion criteria are as follows: (i) recent use of steroids (< 2 months) or other experimental drugs (< 3 months); (ii) use of anticoagulants; (iii) probiotic use; (iv) pregnancy or breastfeeding; (v) known or suspected hypersensitivity to pomegranate fruit or juice; and (vi) serious comorbidities (e.g., hepatic, renal or cardiovascular disease; bleeding disorders; mental and neurological disorders; severe eating disorders, especially anorexia nervosa and bulimia nervosa) [32].

### Data and sample collection

On-site recruitment during IBD patients’ routine follow-up visits by the gastroenterologists is used as a preferential approach. Subjects are also involved through identification in the IBD patient database followed by a phone call to plan a routine-care visit. Patients are screened at the Gastroenterological Unit of St. Orsola-Malpighi Hospital (Bologna, Italy). Participants must give written informed consent to participate in the study. To ensure participant retention, advanced visit scheduling and consistent follow-up through email and text message reminders are performed by a research team member throughout the trial.

Peripheral blood samples are collected in a BD Vacutainer® K_2_ EDTA tube (Becton, Dickinson and Company, Franklin Lakes, NJ, USA) at allocation (t_0_) and after 12 weeks of intervention (t_2_) to isolate peripheral blood mononuclear cells (PBMCs) for transcriptomic analysis [33] and plasma for measurements of inflammatory markers and TMAO and assessments of pomegranate ET-derived metabolites. Plasma is separated by centrifugation for 10 min at 1300×*g* using a refrigerated centrifuge without brake. PBMCs are isolated by SepMate™ tubes (STEMCELL Technologies, Vancouver, BC, Canada) with Lymphoprep™ as a density gradient medium [34]. Urine samples are also collected at the same time points for evaluations of TMAO and pomegranate ET-derived metabolites. Plasma, PBMCs, and urine samples are stored at − 80 °C until analysis.

Stool samples for FC dosage are collected within 24 h before each endoscopy and stored at 2–8 °C until they are assayed. Patients undergo an endoscopic examination at allocation (t_0_) and after 12 weeks of intervention (t_2_). Two biopsies are taken from the sigmoid colonic mucosa (up to 20 cm from the anal verge) at each time point. Biopsies are immediately stabilized in RNAlater® (Thermo Fisher Scientific Inc., San Jose, CA, USA) for transcriptomic evaluations or stabilized by formalin fixation for histological assessments.

Dietary habits of subjects are evaluated during enrolment through a semiquantitative food frequency questionnaire (FFQ) for the assessment of dietary total antioxidant capacity [35]. In addition, food intakes are assessed with a self-administered 3-day dietary record, which is collected by subjects during three consecutive days prior to each sampling day of the intervention period.

Compliant patients are those who consumed at least 80% of the beverages without interruption of the protocol for more than 14 consecutive days.

### Fecal calprotectin dosage

FC is used as a surrogate marker of mucosal inflammation to predict the risk of relapse [36]. The quantification of FC is carried out using the immunochromatographic assay CalFast (Eurospital, Trieste, Italy) according to the manufacturer’s protocol [37, 38]. FC values ≥ 100 μg/g are considered to be predictive of mucosal endoscopic activity as previously demonstrated [9–11].

### Endoscopic and histological evaluation

According to the Mayo endoscopic subscore, a cutoff ≥ 1 is used to discriminate the presence of endoscopic inflammation [39] at baseline (t_0_) and after the intervention (t_2_). Histological activity is scored according to the Geboes grading system [40]. A Geboes cutoff score ≥ 3.1 is assumed to define active histological inflammation [41]. When biopsies show different degrees of activity, the highest degree of inflammation is considered.

### Inflammatory markers

Routine blood tests (e.g., C-reactive protein (CRP), erythrocyte sedimentation rate (ESR)) are performed to evaluate disease activity [42, 43].

Selected marker indicators of systemic inflammation—interleukin (IL)-1β, IL-6, IL-8, IL-10, and tumor necrosis factor α (TNF-α) [42, 44]—are measured on plasma samples by a Human High Sensitivity Cytokine Kit (R&D Systems Inc., Minneapolis, MN, USA) using a Bio-Plex® MAGPIX™ Multiplex Reader (Bio-Rad Laboratories, Hercules, CA, USA). Cytokine levels above the assay’s limit of quantification are also assayed using AlphaLISA specific kits (Perkin Elmer Inc., Waltham, MA, USA). These analyses are performed with baseline plasma before (t_0_) and after the intervention (t_2_).

### Trimethylamine *N*-oxide

TMAO is an amine oxide generated from the dietary lipid phosphatidylcholine by gut microbial metabolism and is a novel marker of cardiovascular risk [45] with potential for assessing disease activity in IBD [46]. It is quantified by UHPLC-MS^n^ in baseline plasma and urine samples at allocation (t_0_) and after the intervention (t_2_). Samples are centrifuged and the supernatants analyzed using the UHPLC DIONEX Ultimate 3000 equipped with a TSQ Vantage triple quadrupole mass spectrometer (QqQ-MS/MS; Thermo Fisher Scientific Inc.) fitted with a heated-electrospray ionization source (H-ESI-II; Thermo Fisher Scientific Inc.) as previously described [47].

### Transcriptomic evaluations

Total RNA is isolated from biopsies using the RNeasy Mini Kit (Qiagen, Hamburg, Germany) according to manufacturer’s protocol. Quantitative real-time PCR (qPCR) analysis is performed to evaluate the expression of key regulator genes of mucosal inflammation in IBD [48, 49] using the CFX Connect™ Real-Time PCR Detection System (Bio-Rad Laboratories, Hercules, CA, USA).

Total RNA is extracted from PBMC pellets using the Quick-RNA™ Whole Blood kit (Zymo Research Corporation, Irvine, CA, USA) according to the manufacturer’s instructions. The gene expression profile is analyzed by whole human genome microarray technology (Agilent Technologies Inc., Santa Clara, CA, USA). qPCR analysis is performed for selected differentially expressed mRNAs to validate the microarray data according to Derlindati et al. [50].

Transcriptomic analyses are performed at baseline (t_0_) and at 12 weeks (t_2_).

### Pomegranate ET-derived metabolites

The determination of pomegranate ET-derived metabolites is performed in plasma samples before (t_0_) and after the intervention (t_2_). Urinary excretion is also estimated. Plasma samples are extracted according to Mena et al. [51], whereas urine samples are centrifuged, diluted, and filtered. Samples are subjected to UHPLC-MS^n^ analysis (linear ion trap MS for identification and triple quadrupole MS for quantification purposes).

### Sample size calculation, randomization, and statistical analysis

The sample size was calculated considering the primary outcome, i.e., the changes in the FC levels at week 12 compared with baseline. Fifty points of FC from the baseline value in at least 70% of patients in the pomegranate juice group and 40% of patients in the placebo group was used for sample size calculations. To detect a significant difference between the two groups (0.05 significance level, two-sided Fisher’s exact test) with 80% power, the minimum number of patients to be enrolled is estimated at 30 in a 2:1 ratio [52]. Presuming a 16% drop out rate, it is necessary to enroll 36 patients (24 subjects assigned to the pomegranate juice group and 12 to the placebo group).

A randomization list is generated using a parallel randomized block design by Random Allocation Software (version 1.0.0) [53], where the participants are randomly allocated in permuted blocks to one of the two groups [54]. This list is made by a colleague who is not involved in subject enrolment and is blinded to the participants, investigators/health care providers, or persons assessing outcomes. The list randomization is placed in sequentially numbered and sealed envelopes; the investigator opens the envelopes in sequence on the day of enrolment.

Intention-to-treat analysis is used to handle participant data. Before any comparison is performed, the normality of the primary outcome is evaluated to choose the most appropriate statistical test. If the data are normally distributed, they are expressed as the mean ± standard deviation and analyzed using general linear models for repeated measures with post hoc comparisons. If the data are not normally distributed, they are reported as median and interquartile range, and the Friedman test, with post hoc pairwise comparisons (Wilcoxon test and Sign test), is performed. The same considerations are applied equally to selected secondary outcomes (changes in circulating inflammatory markers and TMAO levels, changes in pomegranate ET-derived metabolites, transcriptomic changes in intestinal biopsies). A gene network-based approach is used for integrating the quantitative traits (FC and TMAO values, levels of inflammatory markers) and the transcriptomic changes in PBMCs [55]. Additionally, subgroup analysis is performed to assess potential sources of heterogeneity separately as a result of the disease type (UC, CD). This analysis strategy is employed for the primary outcome.

### Confidentiality of data

Personal identity and all personal medical information of the subjects are confidential. Each participant is assigned a unique ID number. Intervention and sample codification are hidden to both investigators and patients (double-blind study) and kept exclusively by the PI. The study participants’ consent is obtained prior to the trial. All biological specimens are to be destroyed after analysis, as indicated in the informed consent given to the subjects and according to the procedures of the Ethics Committee of St. Orsola-Malpighi Hospital (Bologna, Italy).

## Discussion

Currently, there is an increased incidence of IBD, such as UC and CD. IBD clinical symptoms (diarrhea, abdominal pain, and gastrointestinal bleeding) are characterized by relapses and remissions; a severe long-term complication of chronic inflammation is the development of colorectal cancer [56]. First-line therapies in IBD are medications (e.g., 5-aminosalicylates, corticosteroids, immunomodulators, and biologics), which can have severe adverse side effects. The maintenance of IBD remission is a clinical challenge and a relatively neglected area of current research. Several authors have indicated the importance of diet for managing the course of IBD in patients, demonstrating the need for more intervention trials [57, 58].

A strong evidence base suggests that pomegranate fruit exerts anti-inflammatory effects that may alleviate the symptoms of IBD; colon tissue damage, antioxidant status, and inflammation were all ameliorated by pomegranate fruit supplementation in rodent models of IBD [22–25]. Since pomegranate bioactive molecules, mainly ETs, are poorly absorbed in the stomach and small intestine and metabolized mainly in the colon to ellagic acid and urolithins, where they can be found at relatively high concentrations, their effects on intestinal inflammation merit attention. Some work has described the beneficial effects of pomegranate ET metabolites on inflammation in cultured colon fibroblasts [59, 60]. To date, however, no clinical trials in humans have tested the possible role of the consumption of pomegranate juice in the reduction of inflammation in IBD patients with a high risk of relapse.

The effects of the consumption of pomegranate juice in the active group are compared to those of the consumption of a placebo, which has comparable calories and sugar content, in the control group. The use of beverages as an intervention appears to be robustly acceptable and feasible [61]; in addition, the consumption of 100% fruit juice is not associated with an increased risk of diabetes [62]. Consumption of ET-containing foodstuffs and supplements is restricted throughout the trial to standardize the ET daily intake and to minimize influences of other ET sources on the study outcomes. Adherence to dietary restrictions before sample collection is assessed by food diaries, whereas FFQs are used to evaluate exposure to dietary antioxidants over time [35].

Interindividual variability in the production of different ET-derived metabolites [63, 64] is assessed in plasma and urine samples to more clearly define the effects of pomegranate juice and provide a scientific rationale for its consumption. In addition to clinical, biochemical, and endoscopic evaluations, in-depth mechanistic analyses are necessary to provide scientific evidence for the actual cause–effect relationship between pomegranate juice consumption and its possible benefits against inflammatory conditions at both colon and systemic levels. To date, very few trials have studied the associations between nutritional exposure to ET-rich foods and gene expression changes in human tissues of IBD patients [65, 66]. Some investigations were performed in the colon tissues of patients affected by colorectal cancer upon supplementation with pomegranate extracts [67–69].

In conclusion, IBD arises from inappropriate activation of the mucosal immune system, resulting in a state of chronic inflammation. This study is an attempt to bridge the gap between pomegranate consumption and potential amelioration of the inflammatory response. The possibility of preventing relapses or at least minimizing their severity with a dietary intervention would greatly help both physicians and IBD patients.

## Trial status

Recruitment started in December 2016, using protocol version 2 released on July 18, 2016; it is concluded in January 2019.

## Additional file

Additional file 1: SPIRIT 2013 checklist. (DOCX 49 kb)

## Abbreviations

CD: Crohn’s disease
CDAI: Crohn’s disease activity index
CRP: C-reactive protein
cv: Cultivar
EDTA: Ethylenediaminetetraacetic acid
ERK: Extracellular signal-regulated kinase
ESR: Erythrocyte sedimentation rate
ET: Ellagitannin
FC: Fecal calprotectin
FFQ: Food frequency questionnaire
IL: Interleukin
JNK: c-Jun N-terminal kinase
mRNA: Messenger RNA
NF-κB: Nuclear factor κ light-chain-enhancer of activated B cells
PBMC: Peripheral blood mononuclear cell
PI: Principal investigator
qPCR: Quantitative real-time PCR
SCCAI: Simple clinical colitis activity index
STAT3: Signal transducer and activator of transcription 3
TMAO: Trimethylamine *N*-oxide
TNF-α: Tumor necrosis factor α
UC: Ulcerative colitis
UHPLC-MS^n^: Ultra-high-pressure liquid chromatography-mass spectrometry
